# Clinicopathological aspects and proviral load of adulthood infective dermatitis associated with HTLV-1: Comparison between juvenile and adulthood forms

**DOI:** 10.1371/journal.pntd.0008241

**Published:** 2020-04-24

**Authors:** Lucca Santos Souza, Thadeu Santos Silva, Maria de Fátima Paim de Oliveira, Lourdes Farre, Achiléa Lisboa Bittencourt

**Affiliations:** 1 Faculty of Medicine, Federal University of Bahia, Salvador, Bahia, Brazil; 2 Department of Dermatology, Professor Edgard Santos Teaching Hospital, Federal University of Bahia, Salvador, Bahia, Brazil; 3 Laboratory of Experimental Pathology, Gonçalo Moniz Institute, Oswaldo Cruz Foundation (IGM/FIOCRUZ), Salvador, Bahia, Brazil; 4 ProCURE Program, Catalan Institute of Oncology, Bellvitge Biomedical Research Institute (IDIBELL) l’Hospitalet de Llobregat, Barcelona, Catalonia, Spain; 5 Department of Pathology, Professor Edgard Santos Teaching Hospital, Federal University of Bahia, Salvador, Bahia, Brazil; Institute of Tropical Medicine Antwerp, BELGIUM

## Abstract

**Background:**

Infective dermatitis associated with human T-cell lymphotropic virus type-1 (HTLV-1), (IDH), is a chronic eczema occurring in HTLV-1 infected children. Rare cases of adulthood IDH have been reported and no study until now aimed to compare juvenile and adulthood IDH.

**Methodology/Principal findings:**

Twelve cases of adulthood IDH followed for a mean time of 7.5 years were analyzed according to clinicopathological and molecular aspects, comparing them to juvenile IDH cases. Diagnosis was based on the modified major criteria used for juvenile IDH. Proviral load (PVL) assessment was performed by real-time PCR technique. Adulthood IDH presented similar clinicopathological and molecular aspects compared to juvenile IDH. The morphology of lesions and areas of involvement were similar, except for the involvement of the ankles and inframammary folds in the adulthood form. HTLV-1 associated myelopathy/tropical spastic paraparesis (HAM/TSP) occurred in six adulthood IDH patients, with almost equal frequency. However, at least in two patients, HAM/TSP appeared prior to IDH, differently from what was observed in juvenile IDH.

**Conclusions/Significance:**

Adulthood IDH is similar to juvenile IDH according to clinicopathological aspects and PVL levels. Therefore, the same modified major diagnostic criteria for juvenile IDH can be applied to both forms.

## Author summary

HTLV-1 infection is most prevalent in developing countries, and in Brazil, it is most prevalent in poor black communities. The majority of infected people are asymptomatic, but a minority can develop adult T-cell leukemia/lymphoma, HTLV-1 associated myelopathy/tropical spastic paraparesis (HAM/TSP) and/or infective dermatitis associated with HTLV-1 (IDH), the subject of this study. IDH is an exudative chronic eczema that always involves the scalp and retroauricular regions and is characterized by its prompt response to antimicrobial therapy. First described in Jamaican children in the sixties, only in 2001 adulthood onset of IDH was reported, and since then little literature has been produced concerning it. Thus, we aimed to provide a thorough description of this relatively new subtype of IDH, investigating its clinical, pathological and molecular aspects, comparing them to juvenile IDH. Apart from minor differences, such as involvement of extra body sites and earlier onset of HAM/TSP, adulthood IDH is very similar to its juvenile counterpart, and the same diagnostic criteria can be applied to both.

## Introduction

Infective dermatitis associated with the human T-cell lymphotropic virus type-1 (HTLV-1), (IDH) is a chronic and severe form of childhood eczema characterized by an exudative and infected dermatitis, always involving the scalp and retroauricular regions. The majority of IDH patients have been described in Jamaica and Brazil [1,2]. IDH is unknown by most of the health professionals. IDH is frequently associated with HTLV-1 associated myelopathy/tropical spastic paraparesis (HAM/TSP), a myelopathy of insidious onset characterized by bladder disturbances, mild sensory involvement and slowly progressive spastic paraparesis [2]. Progression of IDH to juvenile HAM/TSP has been observed in 54% of the juvenile IDH cases [3]. In Bahia, Brazil, familial clustering of IDH and HAM/TSP has been found in 15 families, 93% in two generations [4].

Since 2001, 11 cases of adulthood IDH have been published, all in Latin America, four of them associated with HAM/TSP [5–9]. However, little is known about adulthood IDH, with no studies showing substantive follow-up of the patients. In the current study, we analyzed clinicopathological aspects and proviral load (PVL) of 12 adult IDH patients followed-up for up to 18.7 years, comparing the results with the same aspects of juvenile IDH observed in Bahia, Brazil, in the same Dermatology service [2]. Clinical aspects at disease progression were also described.

## Methods

### Ethics statement

The study was approved by the Institutional Review Board of the Professor Edgard Santos Teaching Hospital of the Federal University of Bahia, Brazil (CAAE: 91109418.6.0000.0049) and the participants gave written informed consent.

### Study population

The studied sample consisted of 12 patients diagnosed with adulthood IDH between 2002 and 2016 at the Dermatology clinic of the Professor Edgard Santos Teaching Hospital, Brazil. Data were collected at medical appointments and from the patient’s medical records. For comparative purposes, clinicopathological aspects of a group of 42 previously published juvenile IDH patients were taken into consideration [2]. We also included data of 20 juvenile IDH cases for pathological comparison [10] and a group of 18 previously diagnosed juvenile IDH patients for comparison of the PVL levels [11].

### Diagnosis

HTLV-1 infection was detected by ELISA and confirmed by Western blot. Adulthood IDH was diagnosed according to the modified major diagnostic criteria for juvenile IDH (Table 1) [1, 2] in patients whose disease began with ≥19 years of age. Disseminated disease was considered in patients with simultaneous involvement of the scalp, neck, trunk, and limbs [2].

Remission of IDH was considered when an off-treatment patient was free of disease for at least six months [2]. HAM/TSP was diagnosed according to Osame et al. criteria [12].

**Table 1:** Modified major diagnostic criteria for juvenile IDH [2].

1. Presence of erythematous-scaly, exudative, and crusted lesions of the scalp, retroauricular areas, neck, axillae, groin, paranasal and perioral skin, ears, thorax, abdomen, and other sites.
2. Crusting of nostrils.
3. Chronic relapsing dermatitis with prompt response to appropriate therapy but prompt recurrence on discontinuation of antibiotics.
4. Diagnosis of HTLV-1 infection (by serological or molecular biological testing).
Of the 4 major criteria, 3 are required for diagnosis, with mandatory inclusion of 1, 3, and 4. To fulfill criteria 1, involvement of ≥ 3 of the sites is required, including involvement of the scalp and retroauricular areas. HTLV-1: human T-cell lymphotropic virus type 1. IDH: infective dermatitis associated with HTLV-1

IDH—Infective dermatitis associated with HTLV-1

### Exams

Physical, dermatological and neurological examinations and routine laboratorial exams were performed and the patients were also submitted to bacteriological skin cultures and serological tests for HIV, B and C viral hepatitis and syphilis. All patients, except one, were submitted to skin biopsy for pathological and immunohistochemical studies with at least the markers: CD3, CD4, CD8, CD20, CD30 and Ki-67. The follow-up intervals were calculated as to the date of the first visit to the date of the last visit or death (cut-off date: July 2019).

### Treatment

The patients were treated with systemic sulfamethoxazole-trimethoprim (SMX/TMP), 400 mg q12h of SMX and 80 mg q12h of TMP for 15 days, and thereafter received a one-half dose until the disease was controlled, interrupting the drug one month after the lesions have disappeared. Antihistamine drugs, topical corticosteroids and emollients were also prescribed [13].

### Proviral load analysis

Peripheral blood samples of each participant were collected (15ml in EDTA) at diagnosis for PVL determination which was performed as described before from isolated peripheral blood mononuclear cells (PBMC) [11].

### Statistical analysis

GraphPad Prism 5.02 was used to analyze and plot patients’ data. Demographic and PVL data were expressed in median with range. The Mann-Whitney U test was used to compare data between two independent groups. P-values < 0.05 were considered statistically significant.

## Results

### Epidemiological profiles

The studied sample consisted of ten female (83.3%) and two male patients (16.7%). All patients, except one, were African descendants and came from underprivileged socioeconomic backgrounds. Eleven were from Bahia, Brazil, and one was from Pará, a state in North Brazil.

The median age at IDH diagnosis was 44.55 years (range: 25–80 years) and the median age at IDH onset was 36.50 years (range: 24–60 years). Eleven patients were followed-up for a median time of 6 years (range: 2–18 years). Ten patients informed that they never were submitted to blood transfusion. In respect to breastfeeding, only seven were able to give this information and all of them were breastfed. In only one patient it was possible to text for HTLV-1 serology her mother and her mother was negative. Therefore, as she had never been transfused and she was not a drug user, we can assume that in this patient the infection was acquired by sexual transmission (S1 Data). One female patient with IDH and HAM/TSP had only one child who presented the same HTLV-1 associated diseases.

### Clinical profiles

**Fig 1:**
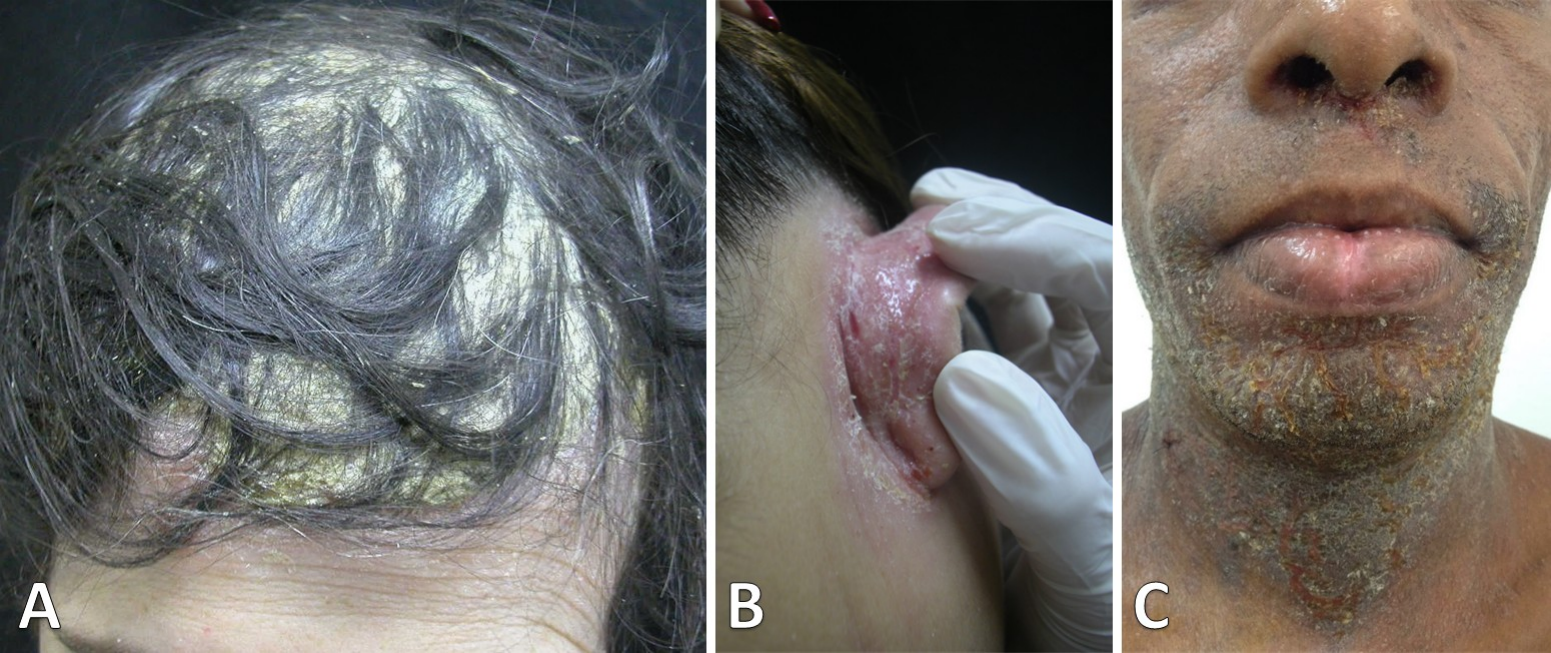
Adulthood IDH skin lesions. (A) Scalp covered by thick and yellow adherent crusts. (B) Erythematous-scaly lesion with fissures in the retroauricular region. (C) Extensive lesion showing involvement of the neck, chin, perioral region and presence of crusts on the nostrils. Fissures on the neck can also be observed.

**Fig 2:**
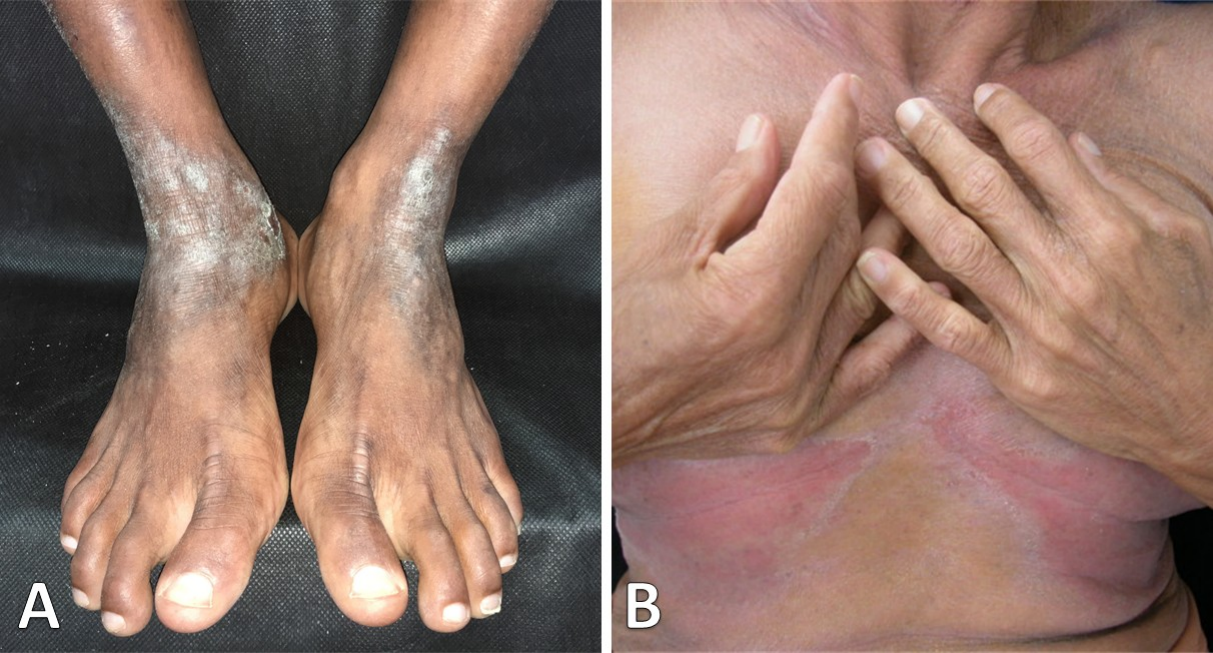
Uncommon adulthood skin lesions. **(A)** Lichenification, hyperchromia and desquamation resembling atopic dermatitis on the ankle and malleolar region. (B) Lesion on the inframammary folds.

The lesions were fetid, erythematous-scaly and exudative, covered by adherent yellowish crusts with mild pruritus. All patients had five or more lesions, including scalp (Fig 1A) and retroauricular areas (Fig 1B), whereas crusting of the nostrils was found in only eight. Five patients presented disseminated disease. In Table 2, in order of frequency, the distribution of the lesions in adulthood IDH is compared with the same data of 42 juvenile IDH patients [2]. Three patients (25%) presented extensive and severe lesions involving contiguous areas. In one of them there was a large lesion, surrounding the lower trunk. Another one presented an extensive lesion on the neck, extending to chin, nostrils, ears and perioral, mandibular and retroauricular regions, with fissures painfully restricting the neck movements (Fig 1C). In the third patient we observed an extensive lesion involving the pubic and genital area, groins and lower abdomen. Few patients have shown lesions in uncommon areas in the anterior aspect of the ankle and malleolar region (Fig 2A) and in inframammary folds (Fig 2B).

**Table 2:** Localization of skin lesions: comparison between adulthood and juvenile IDH.

Areas	Frequency	
	Adulthood IDH	Juvenile IDH [2]
**Scalp**	12 (100%)	42 (100%)
**Retroauricular regions**	12 (100%)	42 (100%)
**Neck**	9 (75%)	37 (88.0%)
**Axillae**	8 (66.6%)	35 (83.3%)
**Groin**	7 (58.3%)	33 (78.6%)
**Paranasal skin**	4 (33.3%)	30 (71.4%)
**Ears**	12 (100%)	30 (71.4%)
**Thorax**	6 (50%)	33 (78.6%)
**Abdomen**	5 (41.6%)	26 (62.0%)
**Antecubital and popliteal fossae**	8 (66.6%)	24 (57.1%)
**Eyelids**	4 (33.3%)	24 (57.1%)
**Forehead**	7 (58.3%)	23 (54.8%)
**Perioral region**	6 (50%)	21 (50%)
**Umbilicus**	3 (25%)	17 (40.8%)
**Limbs**	8 (66.6%)	15 (35.7%)
**External genitalia**	4 (33.3%)	14 (33.3%)
**Buttocks**	2 (16.6%)	7 (16.6%)
**Inframammary fold**	5 (41.6%)	0
**Ankles**	2 (16.6%)	0

IDH–Infective dermatitis associated with HTLV-1

Besides the lesions already described, erythematous-scaly papules, follicular papules, retroauricular fissures and blepharoconjunctivitis were also seen. In Table 3, their frequencies are compared with those of the juvenile IDH patients [2]. Xerosis was found in ten patients (83%) while ichthyosis in only three (25%). Eight patients (66%) complained of pruritus, three of them grading it as intense. Some patients complained of hair loss and two presented small areas of alopecia.

**Table 3:** Frequency of other lesions: Comparison between adulthood and juvenile IDH.

Lesions	Frequency	
	Adulthood IDH	Juvenile IDH [2]
**Erythematous-scaly crusting lesions**	12 (100%)	42 (100%)
**Retroauricular fissures**	7 (58.3%)	32 (76.2%)
**Erythematous-scaly papules**	8 (66.6%)	32 (76.1%)
**Crusting of nostrils**	8 (66.6%)	27 (64.3%)
**Fine papular rash**	2 (16.6%)	25 (59.5%)
**Blepharoconjunctivitis**	5 (41.6%)	24 (57.1%)
**Follicular papules**	4 (33.3%)	19 (45.2%)

IDH–Infective dermatitis associated with HTLV-1

All the laboratorial exams were within normal values, except the serological tests for HIV in one patient and HBC in another. Blood smears were performed in five patients showing flower cells with frequencies ranging from 1% to 2%. Skin cultures performed in seven patients had shown *Staphylococcus aureus*.

### Histopathological and immunohistochemical profiles

**Fig 3:**
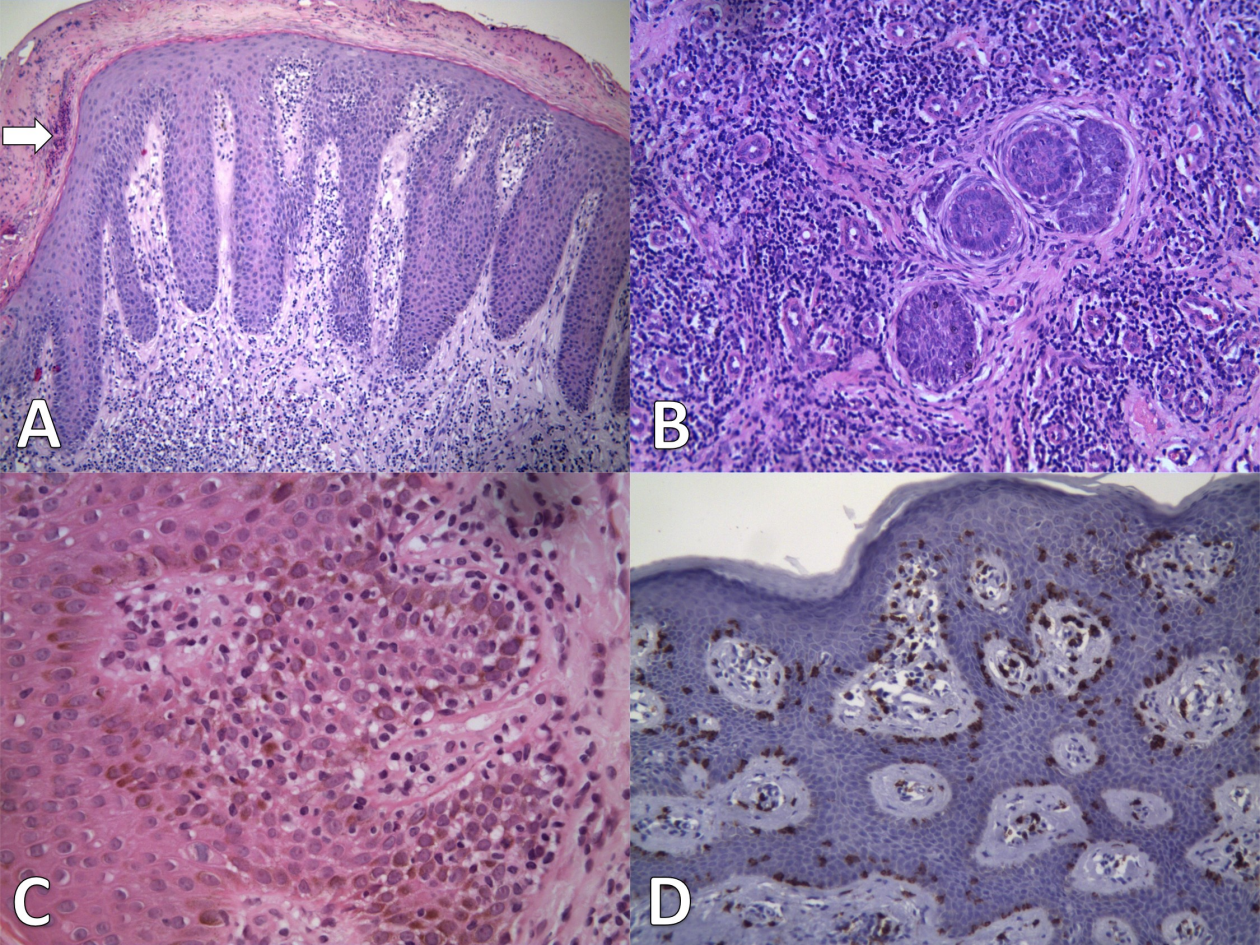
Adulthood IDH immunohistochemical profile. **(A)** Psoriasiform acanthosis, parakeratosis and Munro’s abscess (arrow). Presence of mild infiltration of lymphocytes on the dermis. HE, A 100. (B) Atrophic hair follicles surrounded by fibrous tissue and heavy lymphocytic infiltration. HE, A100. (C) Mild epidermotropism with single lymphocytes arranged along the basal layer. HE, A 400. (D) Acanthosis with presence of CD8+ lymphocytes mainly along the basal layer. CD8 marker, A200. IDH–Infective dermatitis associated with HTLV-1.

All biopsies presented hyper or parakeratosis and acanthosis, in five (41%) with a psoriasiform pattern (Fig 3A). Indeed, three patient had a previous clinical diagnosis of psoriasis and three others of seborrheic dermatitis. Munro’s abscesses were seen in two biopsies and spongiosis in six, varying from mild to moderate intensity. In three, the infiltrate was disposed around hair follicles being of marked intensity in only one biopsy (Fig 3B). Epidermotropism of mild to moderate degree was detected in seven biopsies but Pautrier’s abscesses were not seen. Lining up of single lymphocytes along the basal layer was noted in three biopsies (Fig 3C). Only one biopsy showed lymphocytes with mild atypia. In the dermis there was a superficial perivascular infiltrate of typical lymphocytes, plasma cells and macrophages. All dermal lymphocytes were CD3+ and CD8+. CD8+ cells were also found within the epidermis, sometimes lining the basal layer (Fig 3C). CD4+ lymphocytes were observed in three biopsies but in a lower degree in relation to CD8+ cells. The Ki-67 positivity was less than 5%. The markers CD20 and CD30 were negative.

### Comorbidities

Six patients (50%) also presented HAM/TSP: four had the diagnosis of definite and two of probable disease. The median age at HAM/TSP onset was 46 years (range: 35–71 years). In three of them myelopathy appeared before IDH.

Regarding other viral infections, one patient (8%) had AIDS. One other was HBC-positive, but without symptomatology.

Other comorbidities observed in these patients were: hidradenitis suppurativa, chorioretinitis, strongyloidiasis and crusted scabies.

### Follow-up

The 11 followed-up patients showed a good response to treatment with systemic SMX-TMP but they always presented relapses after therapy withdrawal. Notwithstanding, in the relapses the disease generally appeared less severe and more localized. As sequelae, areas of lichenification or hypochromic patches have been observed. IDH remission happened in only one patient, ten years after IDH onset. Two deaths occurred from unrelated causes.

### PVL results

**Fig 4:**
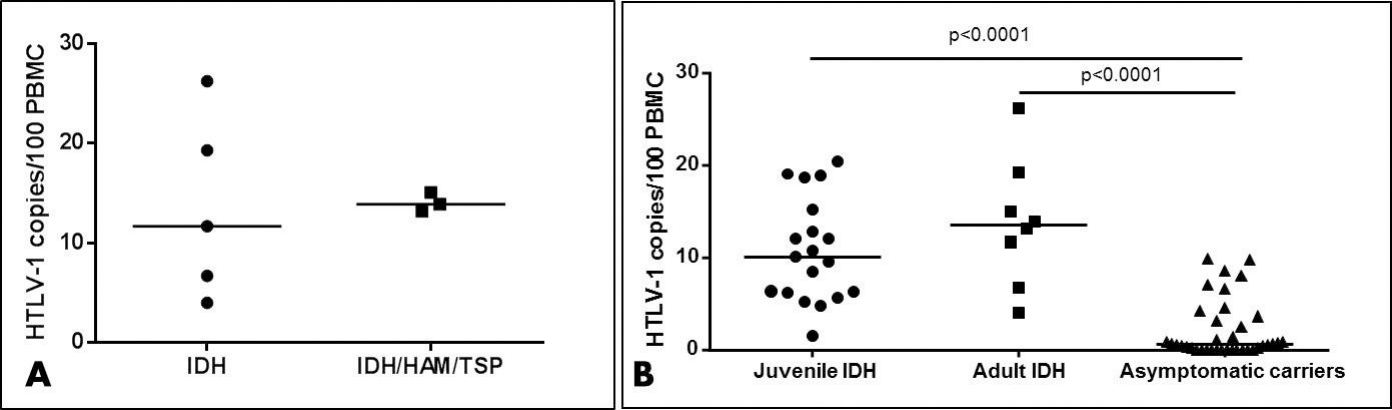
Comparison of HTLV-1 proviral load between different groups. **(A)** The PVL analysis has shown a median of 13.54 copies (4.02–26.22) of HTLV-1/100 PBMC in adulthood IDH. Association of HAM/TSP did not alter PVL significantly (p = 0.7857). **(B)** The PVL of adulthood IDH was significantly higher than that of adult asymptomatic carriers (median of 0.58 copies of HTLV-1/100 PBMC, range (0.10–9.92), p<0.0001) and similar to the PVL of juvenile IDH (median of 10.09 copies of HTLV-1/100 PBMC, range (1.57–20.40), p = 0.2593).

The PVL, evaluated in eight patients (three with HAM/TSP), has shown a median of 13.54 copies of HTLV-1/100 PBMC (range of 4.02–26.22 copies of HTLV-1/100 PBMC). There was no statistically significant difference (p = 0.7857) between the PVL of the IDH group without HAM/TSP, which has a median of 11.69 copies (range of 4.02–26.22) of HTLV-1/100 PBMC, and the IDH group associated with HAM/TSP, which has a median of 13.88 copies (range of 13.19–15.05) of HTLV-1/100 PBMC (Fig 4A). The PVL of the patient with AIDS corresponded to 11.69 copies of HTLV-1/100 PBMC. The PVL of adulthood IDH was significantly higher than that of adult asymptomatic carriers (median of 0.58 copies of HTLV-1/100 PBMC, range of 0.10–9.92, p<0.0001) and similar to the PVL of juvenile IDH (median of 10.09 copies of HTLV-1/100 PBMC, range of 1.57–20.40, p = 0.2593) (Fig 4B).

## Discussion

Like in juvenile IDH, female predominance (82%) also occurred in the adulthood form. The characteristics of the lesions were similar to those found in juvenile IDH. However, differently from the juvenile form, we observed lesions in the ankles and inframammary folds. In the juvenile form, inframammary involvement only occurs when the patient reaches puberty. In three patients extensive lesions were also detected similar to those found in juvenile IDH [2,14]. The frequency of the erythematous scaly-papules, follicular papules, retroauricular fissures and blepharoconjunctivitis was also similar to that described in the juvenile form [2].

The chronic relapsing characteristic was observed in all 11 patients followed-up. Crusting of the nostrils was seen in eight patients, but this aspect is no longer considered as an obligatory criterion for diagnosis [2]. We did not consider the La Grenade minor criteria [1] because they are not essential for diagnosis and can appear in many other dermatoses and/or in asymptomatic HTLV-1 carriers.

Two patients had previous biopsies erroneously diagnosed as psoriasis. Clinical differential diagnosis should aim to rule out seborrheic (SD) and atopic dermatitis (AD), mainly SD because it appears more frequently among HTLV-1 infected than in non-infected individuals [14].

Microscopically, the lesions appear as a spongiotic or chronic dermatitis and cannot be distinguished from other forms of eczema. The presence of psoriasiform acanthosis and Munro’s abscesses can be a confounding factor leading to an erroneous microscopic diagnosis of psoriasis. Although some aspects were suggestive of psoriasis these findings can be observed in other infected dermatoses. In cases of doubt it is important to correlate the microscopical and immunohistochemistry findings with the clinical aspects. The differential diagnosis between psoriasis and IDH is important because the treatment is different between them. It is important to emphasize that the diagnosis of IDH is primarily clinical and the clinical diagnosis between them is not difficult. Considering that IDH may progress to acute adult T-cell leukemia/lymphoma (ATLL) [2] it is important to do a differential microscopic diagnosis with early mycosis fungoides (MF). Although some IDH cases presented epidermotropism resembling early MF, in these cases the lymphocytes were not atypical. Similar aspects can also be observed in benign simulators of MF, including juvenile IDH [10].

Like juvenile IDH, the inflammatory infiltrate is mainly composed of lymphocytes, most of them CD8+, distributed in the dermis and sometimes in the epidermis.

The frequency of HAM/TSP association was high (50%), quite similar to what is observed in the juvenile form (54%) [3]. In contrast with the juvenile form, in which IDH always precedes HAM/TSP [2], in at least two adult patients it appeared after the development of HAM/TSP.

As is known, HTLV-1 infected patients may present more frequently xerosis, ichthyosis, strongyloidiasis and crusted scabies, and these conditions may appear in both forms of IDH [14]. Although IDH patients may infrequently progress to ATLL, in the current study this progression did not occur [2]. Considering familial clustering [4], we found one female patient with IDH and HAM/TSP whose the only child had both diseases.

It was shown that the PVL of adulthood IDH was similar to PVL of the juvenile form and significantly higher than that of adult asymptomatic carriers. PVL was previously reported in six adult IDH patients in two papers with different values [8,9]. In the first paper, three patients presented a PVL of around 4 copies of HTLV-1 in 100 PBMC and the other patient less than 1 copy HTLV-1 in 100 PBMC [8]. In the other paper, one patient presented a PVL less than 1 copy HTLV-1 in 100 PBMC while the other patient presented a PVL of 61 copies of HTLV-1 in 100 PBMC [9]. This differences of results could be related to different methodological procedures. Nevertheless, we would like to highlight that in our study PVL in adulthood IDH was similar to PVL of a group of juvenile IDH of the same endemic area [11]. As considered in the literature, the PVL of the HIV coinfected patient was not different from the median PVL of the group [15].

On the other hand, the association with HAM/TSP did not alter significantly the PVL value in adulthood IDH as observed in the juvenile form [11]. The PVL of the patients with adulthood IDH and HAM/TSP was similar to the PVL of adulthood HAM/TSP patients without dermatologic involvement from the same endemic area [16]. Juvenile IDH and adulthood HAM/TSP present and overproduction of inflammatory cytokines and high levels of PVL, which can be the cause of the inflammatory processes, respectively, in the skin and in the central nervous system [17].

Although the number of patients is small, this paper includes its biggest casuistic. Curiously, in Japan, where HTLV-1 infection is highly endemic [18], there is no reported case of adulthood IDH.

### Conclusion

The comparison between juvenile and adulthood IDH showed similar results in relation to clinicopathological and molecular aspects, association with HAM/TSP and treatment responses. Therefore, we can conclude that the diagnostic criteria employed for juvenile disease is also applicable to adulthood IDH.

## Supporting information

S1 Data Epidemiological data and PVL of 12 adulthood IDH cases. (XLSX)
